# The Institutional Library

**Published:** 1921-01-01

**Authors:** 


### THE INSTITUTIONAL LIBRARY.

**The Principles and Practice of Medicine.** By Sir William Osler, Bart., M.D., F.R.S., late Regius Professor of Medicine at Oxford University, and Thomas McCrae, M.D. Ninth, thoroughly revised, Edition. (New York and London: D. Appleton & Co. Cloth, 30s. net, with portrait.)

Since the first edition of this most valuable text-book was published nearly thirty years have elapsed, years in which enormous strides in medical understanding have been made. The war interrupted its usual triennial appearance in completely revised form, but at the close of hostilities the author turned actively to work on his part of the revision for the present edition, and this was practically completed at the onset of his last illness. As Dr. McCrae explains in his preface, it is a grim coincidence that Osler had planned to give up active participation in the revision when he reached the age of seventy years.

Of so excellent a book it is unnecessary here to express our admiration. Suffice it to say that the modernisation is skilfully and thoroughly done. Many parts have been entirely rewritten, and the whole form largely recast. New sections necessarily embrace Paratyphoid, Focal Infection, Trench Fever, Gas Poisoning, Acidosis, Infectious Jaundice, etc. The matter of precise arrangement and classification of disease is of such obvious and well-known difficulty that one must, in parts, compromise against any rigid plan, but there is no doubt that in the volume before us the choice has fallen on that construction which gives the maximum of assistance, both to the student and the profession in general.

**Functional Mental Illnesses and the Interdependence of the Sympathetic and Central Nervous Systems in Relation to the Psychoneuroses.** By R. G. Rows, M.D., and David Orr, M.D. (Edinburgh: Oliver & Boyd. Pp. 63. Price 3s. 6d.)

The first of the articles reprinted in this brochure formed the Morison Lectures delivered by Dr. Rows at Edinburgh early in this year; the second is by Dr. Orr, and is included "in order to present a more comprehensive view of the various factors which regulate normal mental processes and assist in determining mental illnesses," it having been possible "only to touch lightly on the importance of the endocrinic glands in nervous physiology and pathology." The investigations were made not only among soldiers who had broken down under the strain of war, but information was also derived from cases which occurred in the civilian population.

Drs. Rows and Orr have already done much valuable work in collaboration, and their names attached to any scientific essay are an earnest that much labour and thought have gone to the production. The present one is no exception. Their conclusions are based upon conscientious research, and even those who do not agree with them cannot accuse them of hasty generalisation.

Mental disorder is too frequently considered from the purely "mental" point of view. It is true that "the seat of the processes underlying mental phenomena is to be found in the cortex cerebri"—though even this is apparently not understood or taken into account by those who localise the disorder in a supposititious "mind." But, further, "the interactions between the brain and the organs of the body, as well as those between the organs of the body and the brain, must be understood before we shall be in a position to comprehend and to explain the complex symptomatology of functional mental illnesses." It is necessary to understand how the integration—or building up—of the nervous system takes place by the aggregation of simple reflexes and the evolution of the more complex system of inhibitions or conditional reflexes. Many and various stimuli act on the body and eventually an organism more or less adapted to its environment results. These stimuli may be of a certain character and are called "psychic," while others are spoken of as "physical." The nervous mechanism is delicately adjusted and may easily be put out of gear, more especially if substances such as the internal secretions are diminished or are in excess. Some stimuli of a disturbing nature, with their associated unpleasant emotional correlatives, may dominate, consciously or unconsciously, mental functioning, and until this stream of energy is checked or diverted, the patient is ill. A vicious circle may be established. Morbid mental stimulus or psychic trauma brings about changes in the physiological mechanism of the endocrino-sympathetic system, and this in its turn interferes with the proper functioning of the central nervous system.

This is but a brief sketch of some of the points discussed in these most interesting papers. It may serve to show, however, that the whole question of mental disorder is not quite so simple as some appear to imagine. Drs. Orr and Rows are of opinion that an optimistic position may be taken up as to the future treatment of mental illnesses, provided they are dealt with in the primary stage.

**Handbook for Tuberculosis Workers.** By N. Bardswell, M.D., F.R.C.P. (London: Bale, Sons & Danielsson. 1920. Duodec. Pp. 66. Price 1s. 6d.)

Two sorts of medical literature recent years have made this country very rich in; they are handbooks on sexual pædagogy and handbooks on tuberculosis addressed to the laity. What a young tuberculosis worker ought to know is the theme of this little grey brochure. Sir Henry Gauvain contributes a note on the care of children discharged from surgical tuberculosis hospitals.

**Home Exercises for Spinal Curvatures.** By Richard Timberg, M.R.C.S. (Eng.), L.R.C.P. (Lond.), G.D. (Stockholm), Medical Officer Physico-Therapeutic Department St. Thomas's Hospital. Second and Revised Edition. (London: William Heinemann Medical Books, Ltd.)

Remedial treatment of deformities of whatever kind unless accompanied by carefully selected home exercises may fail to accomplish fully the purpose, for the patient is apt to resume, between the treatments, the posture or habit which contributed to the deformity. The author of these home exercises, when he published the first edition of the work some years ago, addressed himself to the subject in a practical manner, and succeeded in producing a text-book written in popular language which the medical practitioner might usefully pass on to his patients to aid them in the correct execution of such selected exercises as the conditions might indicate. The simple exposition on the construction of the spinal column and the reference to the chief factors contributing to deformities contained in the opening chapters are especially useful, for there can be no surer aid to the correct performance of any exercise than a practical knowledge of its purpose. These chapters might be perused with advantage by all parents. The exercises are lucidly described and well illustrated; their merit lies in their simplicity, and their value has been amply demonstrated. The edition under

**The Institutional Library**—(*continued*).

review includes a chapter dealing with some of the milder degrees of foot deformity, of frequent occurrence in children suffering from spinal curvature. We are glad to note that the author lays stress upon the periodical supervision of the patient.

**Electro-Therapy: its Rationale and Indications.** By J. Curtis Webb, M.A., M.B., B.C. (Cantab). (Churchill. Price 5s. net.)

The object of this little book is the concise description of the "modern view as to the action on the human body of each of the forms of electric currents used in medicine and the indication of the various ailments to which they are applicable." It is written for those members of the medical profession who, though not engaged upon the actual application of this method of treatment, are yet anxious (and these should include all practising physicians and surgeons) to know *when* such means are indicated. The book contains, therefore, nothing in the way of elaborate descriptions of technique, but in simple language it states the principal facts relating to continuous and interrupted, high-frequency and diathermy, currents; static electricity; radium and *x*-rays. In Part II. there is given a comprehensive list of diseases in which it is reasonable to apply electrotherapy. The cardinal symptoms of the various affections are stated. In malignant disease the author is insistent that, where operation is possible, it should be followed by raying treatment, and that electro-therapy should never be used alone save in inoperable cases. Undoubtedly there seems to be a very important future for this branch of science, and we can heartily recommend the book to all who wish to know the extent of its present sphere of usefulness.

**The Prevention and Destruction of Rats.** By Elliot B. Dewberry. With a Preface by Sir A. E. Shipley, F.R.S., Sc.D. (London: John Bale, Sons & Danielsson, Ltd. Price 2s. net.)

This is one of the most practical of the small books upon rat destruction which we have had opportunity of reading. The author writes from personal experience and makes an exhaustive list of rat-catching methods and contrivances. A useful point, and an essential one in that it is so often missed, is the fact that full references and names are given, enabling the reader easily to obtain the various traps and apparatus mentioned. Most of us know the danger of the rat scourge, but few know how to catch these pests in quantities. The author provides that information and in easily readable form.

**A Synopsis of Surgery.** By Ernest W. Hey Groves. (Bristol: John Wright & Sons, Ltd. Price 17s. 6d. Fifth Edition.)

The concise descriptions and orderly arrangement of this book have for same years past been of the greatest assistance to students in cataloguing knowledge gained from experience in the wards and from the more exhaustive text-books on surgery.

By bringing the work up to date, especially in respect of experience gained in war surgery, the author has given to the busy practitioner an opportunity to keep in touch with this ever-advancing science.

**Selected Lectures and Essays.** By Sir John Bland Sutton. London: Wm. Heinemann. (Medical Books, Ltd.)

The fourth edition of this interesting publication is well produced, and the collection of most interesting and varied papers it contains cannot fail to attract a yet larger circle of readers. Collected and presented to the medical world as a reminder of the many hours which the author spent with his "old students," they still form, although most have been published elsewhere, a delightful and fascinating volume.

**Notes and Suggestions on Dysentery, Trench Fever, Gas Poisoning, in Relation to Disabled Ex-Service Officers and Men.** Prepared for the Ministry of Pensions. (The Stationery Office. Price 6d. net.)

This pamphlet is the work of four collaborators, and presents a special aspect of the three conditions of which it treats—namely, the sequelæ and after-effects in general of the acute early forms (which are briefly summarised, but not discussed in full). As a sensible practical guide to medical boards and medical referees, free from red-tapiness, and scientifically all that can be desired, the pamphlet occupies a place of its own, and offers a kind of help that cannot be obtained from any other publication. It is stated to be a joint effort of the Ministry of Pensions and the Ministry of Health, and it reflects credit on both bodies and on their responsible officials.

**Guide to Industrial Welfare Work for Beginners and Others.** By Constance Ursula Kerr, LL.D. (Hons.), Welfare Supervisor C.W.S. Works, Irlam, nr. Manchester. (Published by the Co-operative Press Agency, 1 Balloon Street, Manchester. Price 2s.)

Welfare work is becoming so intimately incorporated with industry as to be regarded a necessary and essential equipment where large communities of workers are gathered. Its essence of success lies entirely in the hands of the welfare worker to whose technical knowledge and competence is added the virtue of tact. Miss Kerr displays with skilful experience the value of this virtue and its usefulness in avoiding or overcoming the manifold difficulties which beset the path of the welfare supervisor. The chief value of her book is the manner in which welfare operations can be established in a factory of any dimensions with complete and mutual reciprocation between employers and employees. She particularly aims at eliminating the mistake of regarding the welfare worker as a charitable installation, or an act of philanthropy without profit, but, with full co-operation and a corresponding improvement in personnel and output, the presence of the welfare worker can be abundantly justified. The subject of welfare is a large one, and its successful and wise administration a profound study to those who would adopt it. There is much practical advice and help to be gleaned from this "Guide to Welfare Work," and it is to be thoroughly recommended.

**A Pocket-Book of Ophthalmology.** By A. J. Ballantyne, M.D. (Livingstone. 1920. Pp. 119. Price 5s. 6d. net.)

This is an elementary text-book for the student of medicine, interleaved throughout with blank pages upon which he may enter notes at discretion. The teaching is well arranged and clear, and should very materially help the student in mastering what for want of simple short text-books such as this appears to him too often a complicated and bewildering branch of surgery. Since in (general) practice by far the commonest ocular lesions which confront the medical man are injuries caused by foreign bodies, the few cursory words in which this portion of the subject is dismissed might well be expanded in a future edition.

